# The adaptor protein p62/SQSTM1 in osteoclast signaling pathways

**DOI:** 10.1186/1750-2187-7-1

**Published:** 2012-01-04

**Authors:** Stephen McManus, Sophie Roux

**Affiliations:** 1 Division of Rheumatology, Faculty of Medicine, University of Sherbrooke, 3001, 12th Avenue North, Sherbrooke, PQ, Canada

**Keywords:** osteoclast, p62, RANKL signaling, autophagy

## Abstract

Paget's disease of bone (PDB) is a skeletal disorder characterized by focal and disorganized increases in bone turnover and overactive osteoclasts. The discovery of mutations in the *SQSTM1/p62 *gene in numerous patients has identified protein p62 as an important modulator of bone turnover. In both precursors and mature osteoclasts, the interaction between receptor activator of NF-κB ligand (RANKL) and its receptor RANK results in signaling cascades that ultimately activate transcription factors, particularly NF-κB and NFATc1, promoting and regulating the osteoclast differentiation, activity, and survival. As a scaffold with multiple protein-protein interaction motifs, p62 is involved in virtually all the RANKL-activated osteoclast signaling pathways, along with being implicated in numerous other cellular processes. The p62 adaptor protein is one of the functional links reported between RANKL and TRAF6-mediated NF-κB activation, and also plays a major role as a shuttling factor that targets polyubiquitinated proteins for degradation by either the autophagy or proteasome pathways. The dysregulated expression and/or activity of p62 in bone disease up-regulates osteoclast functions. This review aims to outline and summarize the role of p62 in RANKL-induced signaling pathways and in ubiquitin-mediated signaling in osteoclasts, and the impact of PDB-associated p62 mutations on these processes.

## Background

Encoded by *SQSTM1*, the ubiquitin-binding protein p62 or sequestosome 1 is a scaffold and an adaptor protein that modulates protein-protein interactions, and as major component of multiprotein complexes, it mediates various cell functions, including cell signaling, receptor internalization, protein turnover, and gene transcription [1]. Mutations of the *SQSTM1 *gene have been detected in a high proportion of patients with Paget's disease of bone (PDB), thus highlighting the critical importance of p62 in the regulation of bone physiology [2]. While the most clearly established function of p62 is its role as a scaffold protein for intracellular signaling and the selective activation of NF-κB [1,3], p62 also plays a major role as a shuttling factor that targets polyubiquitinated proteins for degradation by either the autophagy or proteasome pathways [4,5].

### Bone remodeling and osteoclast function

Bone remodeling is constant and dynamic, with a balance maintained between bone resorption and subsequent new bone formation. The cells responsible for these interrelated processes include the bone-resorbing cells, i.e. osteoclasts, which are derived from hematopoietic cells, and bone-forming cells, i.e. osteoblasts, which are of mesenchymal origin. Skeletal homeostasis depends on maintaining tight control of the number of osteoclasts active at any site [6]. Accelerated or increased bone resorption may involve elevated osteoclastogenesis from precursor cells, an increase in the fusion and/or activation of osteoclasts, and the prolongation of their lifespan via the inhibition of osteoclast apoptosis [7,8]. Osteoblasts or stromal cells support osteoclast differentiation and activation, and these processes are regulated by two signaling pathways, which are activated by M-CSF and receptor activator of NF-κB ligand (RANKL) respectively, and an ITAM (immunoreceptor tyrosine-based activation motif)-mediated co-stimulatory signaling [9]. RANKL is a membrane-bound, TNF-related factor expressed by osteoblast/stromal cells and activated lymphocytes, and it can be cleaved to produce a soluble form. *In-vitro *and *in-vivo s*tudies have clearly shown that RANKL, by binding to its membrane-bound receptor RANK, plays a crucial role in the formation, survival, and bone-resorbing activity of osteoclasts [10-12]. The fine-tuning of bone resorption also involves osteoprotegerin (OPG), a secreted decoy receptor of the TNF receptor family expressed by osteoblast/stromal cells, but also by many other cells in the bone marrow microenvironment. OPG recognizes RANKL, and therefore competes with RANK and leads to the inhibition of osteoclast differentiation and bone-resorption [13]. After carrying out resorption, osteoclasts undergo apoptosis. Ordinarily, osteoclasts have been shown to be sensitive to apoptosis induction by a number of cytokines and factors, including Fas-ligand, TRAIL, and TGFβ [14-16].

### The p62 scaffold, an adaptor protein with multiple binding domains

**Figure 1:**
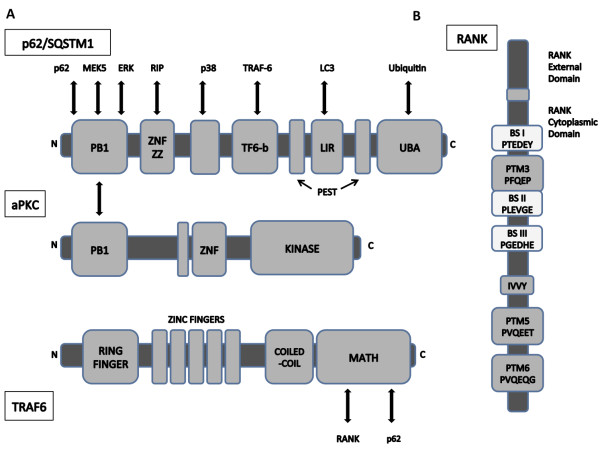
**Interaction motifs and domains of RANKL signal intermediaries**. A: *p62 and an aPKC*. The cytosolic p62 protein, encoded by the *SQSTM1 *gene, is a scaffolding protein that interacts with the RANK signaling complex, and is one of the functional links reported between RANKL and TRAF-6-mediated NF-κB activation. Multiple interaction motifs located within p62 enable recruitment of specific proteins and regulation of downstream signaling pathways. RIP binds to the ZZ domain, whereas TRAF6 interacts with the TF6-b sequence, and the aPKC isoforms, ERK, and others interact with the PB1 domain. This PB1 interaction directs the aPKCs in the NF-κB pathway. The UBA domain binds to polyubiquitin chains, and is important for the ubiquitination of TRAF6. PB1, PB1 dimerisation domain; ZZ ZNF, ZZ-type zinc finger; TF6-b, TRAF6 binding sequence; PEST, (P, Proline; E, Glutamate; S, Serine; T, Threonine) rich sequence; UBA, ubiquitin associated; PS, pseudosubstrate region; MATH, meprin and TRAF homology. **B: ***The RANK receptor*. RANK has six known putative TRAF-binding motifs (PTM 1-6) of which three have been involved in osteoclast signaling. TRAF6 binds to membrane-proximal motifs such as PTM3 (PFQEP^369-373^), whereas PTM5 (PVQEET^559-564^) and PTM6 (PVQEQ^604-609^) most likely interact with TRAF2 and TRAF5 [22,23]. In addition, three other TRAF6- binding sites have been identified, BS I (PTEDEY^340-345^), BS II (PLEVGE^373-378^) and BS III (PGEDHE^447-452 ^[25,26].

The p62 primary sequence embodies at least 9 protein-interaction domains with structural motifs including a ubiquitin (Ub)-associated (UBA) domain at its C-terminus, two PEST sequences between which an LC3-interaction region (LIR) stands, a binding site for the RING-finger protein TRAF6, a domain binding p38 as well as LIM-containing proteins, a ZZ finger interacting with RIP, a PB1 domain that binds atypical PKCs (aPKCs), but also ERK, NBR1, MAPKK5 (MEK5), MEKK3, and p62 itself, and an N-terminus capable of direct interaction with the proteasome (26S and S5a subunits) [3,17-19]**(**Figure 1-A**)**. With such numerous protein-protein interaction motifs, p62 is considered a scaffold, serving as the switchboard from which the RANKL activation signal is propagated, and thus playing an important role in the osteoclast.

### The p62 scaffold in RANKL-induced signaling

In both precursors and mature osteoclasts, the interaction between RANKL and RANK results in signaling cascades that ultimately activate transcription factors, particularly NF-κB and NFATc1 [9]. The sequence of activation requires the recruitment of TRAF6, which is responsible for most of the downstream events that lead to osteoclast differentiation and activation [20].

#### P62 as a functional link between RANKL and TRAF6-mediated NF-κB activation

**Figure 2:**
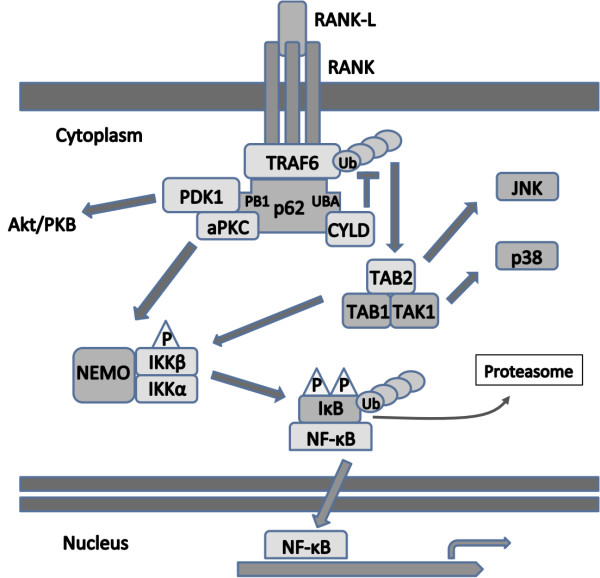
**p62 in osteoclast signaling and protein trafficking**. The binding of RANKL to the receptor protein RANK at the plasma membrane induces the formation of a trimer, triggering the recruitment of a series of adaptor proteins. TRAF6 catalyses Lys63-linked autoubiquitination via intrinsic E3 ubiquitin ligase activity, which is regulated by the UBA domain of p62, and eventually deubiquitinated post-recruitment of CYLD to p62. In the interim, this ubiquitination permits activation of the TAB1-TAB2-TAK1 complex, in turn activating the MAP kinases, as well as NF-κB-inducing kinase (NIK), which leads to phosphorylation and activation of IKKβ. Activation of TRAF6 and p62 also leads to activation of the Akt/PKB pathway. Simultaneously, p62 binds aPKC through its N-terminal PB1, allowing for the phosphorylation of IKKβ by the aPKC. Once activated, IKKβ phosphorylates IκB, which is subsequently ubiquitinated, and degraded through the proteasome system, liberating NF-κB to translocate to the nucleus and interact with transcription promoters.

RANK, like other members of the TNFR family, lacks the capability for intrinsic enzymatic activity through its intracellular domain, and relies principally on TNFR associated factor (TRAF) for signal transduction. The cytoplasmic domain of RANK directly interacts with five of the six TRAF proteins, with different binding sites, that of TRAF6 being more proximal to the membrane [21,22]**(**Figure 1-B**)**. Three RANK TRAF-binding motifs have been shown to be involved in the osteoclast formation and activation, as in the RANK-induced activation of NF-κB and MAP kinases JNK, ERK and p38 [23]. In addition, TRAF6-specific binding sites have also been identified, and TRAF6 signaling appears crucial in osteoclast signaling [24-26]. The binding of RANKL to its receptor RANK results in the recruitment of TRAF6; activated TRAF6 may then stimulate NF-κB activity by activation of the IκB kinase (IKK) complex, either through aPKC or TAK1-dependent phosphorylation, a process that requires NEMO (IKKγ) ubiquitination for optimal activation [27]**(**Figure 2**)**. The p62 scaffolding protein is one of the functional links reported between RANKL and TRAF6-mediated NF-κB activation [1]. Once bound to TRAF6, the p62-TRAF6 complex interacts with atypical protein kinase C (aPKC) proteins resulting in the formation of a multimeric protein complex that regulates NF-κB activation via the phosphorylation of IκB kinase β (IKKβ) [28-30]. The p62 complex also binds the scaffolding receptor interacting protein (RIP) and phosphoinositide-dependent kinase 1 (PDK1), recruiting aPKCs to TNF-α signaling complexes [31,32].

TRAF6 also forms complexes with TGFβ-activated kinase 1 (TAK1) and adaptor proteins TAB1 and TAB2 [33]. When TAK1 is activated, it in turn phosphorylates NF-κB-inducing kinase (NIK), which activates the IKK complex, leading to NF-κB pathway activation. As for the MAP kinases, TAK1 also activates the JNK pathway, while TAB1 recruits and binds p38 to the TRAF6 complex, leading to activation of its pathway [34,35]. In addition, RANKL-induced TRAF6 activates the Akt/PKB pathway through a signaling complex that involves c-Src [36]. All these signaling molecules contribute to osteoclast differentiation, survival and activity [9,20]. As a scaffold, p62 plays a major role in RANK-induced signaling, through the recruitment and formation of signaling complexes, and its ability to bind and activate TRAF6 as described below. Confirming the major role of p62 in the RANK/RANKL signaling pathway, genetic inactivation of *SQSTM1 *leads to the inhibition of IKK and NF-κB activation induced by RANKL, as well as NFATc1 synthesis, and to impaired osteoclastogenesis in mice [28].

#### p62 and PDK1/aPKC activation in osteoclasts

In a murine model, RANKL stimulation has been reported to induce the formation of a ternary complex between TRAF6, p62 and atypical PKCs (aPKCs), identifying p62 as an important mediator during osteoclastogenesis [28]. Consistent with this data, it has also been shown in human osteoclast cultures that following RANKL stimulation, PKCζ, P-PKCζ/λ and P-PDK1 as well were associated to p62 [37]. This is consistent with the knowledge that PKCζ, like other aPKCs, is a substrate of PDK1, indicating a role for PDK1 in p62-aPKC signaling, and perhaps other TRAF6-p62 signaling pathways. Indeed, PDK1, a serine/threonine kinase, is a recognized master protein kinase in the regulation of many cell-signaling pathways, known to be constitutively active, and can be further activated by tyrosine phosphorylation (Tyr^9 ^and Tyr^373/376^). Many proteins involved in the PI-3K signaling pathway, like PI-3K, Akt, and the phosphatase and tensin homolog on chromosome 10 (PTEN) are mediated by PDK1 [38]. PDK1 has also been found to phosphorylate and activate other members of the cAMP-dependent, cGMP-dependent, and protein kinase C kinase families of proteins including both novel and atypical PKCs, p70S6K, and others [39]. However, its exact role in osteoclast p62 signaling remains to be elucidated.

The IKK complex is cardinal to the activation of NF-κB, and is composed of three subunits, one of which is regulatory, called IKKγ or NEMO, and two that are catalytic, IKKα and IKKβ. Notably, in non-osteoclastic cells, p62 has been shown to regulate IKK activation through its ability to bind the atypical PKCs like PKCζ and PKCλ, who favor phosphorylation of IKKβ [29,40]. In addition, in PC12 cells, IKKβ has been shown to be recruited to the multiprotein complex including p62, TRAF6 and aPKC [41].

#### p62 and the TRAF6/CYLD balance

The protein p62 appears to be a molecular adaptor associating not only TRAF6 but also the de-ubiquitinase CYLD [42]. p62 interactions with TRAF6 stimulate TRAF6 K63-linked autoubiquitination and E3 ligase activity, and regulate the synthesis of K63 chains on target substrates. In addition to the UBA domain, the PB1 domain of p62 is needed for TRAF6 polyubiquitination [1]. Ubiquitination of TRAF6 is an important mechanism mediating its signaling functions [43,44]. However, rather than an absolute requirement for TRAF6 E3 ligase activity in mediating downstream signaling, K63-linked ubiquitination of TRAF6 should be viewed as a marker of activation [27]. In addition to its well established role in the activation of TRAF6, p62 may also regulate a de-ubiquitinating enzyme (DUB) with a specificity for Lys^63 ^(K63) chains (CYLD), that interacts with TRAF6, and reverses the processing of protein ubiquitination. The decrease in the activity of CYLD leads to the accumulation of Lys^63 ^(K63)-ubiquitinated substrates [42]. p62 interacts with CYLD and promotes the binding of CYLD to TRAF6, and this molecular interplay requires the C-terminal domain of p62 [45]. CYLD negatively regulates NF-κB activity by reducing TRAF6 autoubiquitination [46,47]. CYLD negatively regulates the activation of IKK and JNK, and its expression is markedly upregulated under conditions of RANKL-induced osteoclastogenesis [45]. Thus CYLD appears to be a crucial down-regulator of RANK signaling in osteoclasts. Accordingly, CYLD-deficient mice display severe osteoporosis linked to aberrant osteoclast differentiation and have larger and more numerous osteoclasts, which are hypersensitive to RANKL [45].

#### NFATc1 and ERK regulation by p62

RANKL specifically and strongly induces the nuclear factor of activated T cells (NFATc1 or NFAT2), a master regulator of osteoclast differentiation [48]. This induction is dependent on TRAF6/NF-κB and c-Fos pathways, as well as calcium signaling. NFATc1 also specifically autoregulates its own promoter particularly during RANKL-induced osteoclastogenesis [9]. Naturally, TRAF6 activity is affected by p62 interaction, affording p62 some measure of indirect control over NFATc1 signaling. However, kinases may also contribute to the nuclear shuttling of this factor, as PKCζ has been shown to interact with NFATc1, and may modulate NFAT-mediated transcription by increasing the activity of its N-terminal transactivation domain [49]. In addition, RANKL also induces MEK/ERK pathway which contributes to osteoclast differentiation and survival [50]. The p62 UBA domain plays an important role in the activation of NF-κB, NFAT and in ERK phosphorylation [51].

### Role of p62 in protein trafficking and turnover

#### P62 and protein turnover

The signal for targeting proteins for degradation by either the autophagy or proteasome pathways is ubiquitination, both processes in which p62 plays a major role through binding ubiquitinated protein and trafficking as well. p62 may be involved in the formation of multimeric protein complexes as a result of the interactions of its PB1 domain with other proteins, and it may also play a role in protein turnover as a result of its UBA domain at its C-terminus, which binds non-covalently to polyubiquitin chains, and finally through its association with the proteasome (26S and S5a subunits) via its N-terminus domain [19]. In a well-characterized neuronal model, p62 has been shown to direct the addressing of protein complexes containing the neurotrophin receptors, TRAF6, and PKCζ, making it an essential player in signaling protein degradation and recycling [52,53].

#### Autophagy and LC3 interaction

Autophagy takes place in all cells, in order to maintain cell homeostasis or in response to stress or starvation, by eliminating damaged components, and providing cells with energy and nutrient resourcing [54,55]. p62 has been identified as an LC3-interacting protein implicated in autophagy [56-58]. Studies have shown that p62, along with ubiquitinated proteins, is transported into autophagosomes, suggesting that p62 is a receptor for these proteins that directs them to lysosomes [56,58]. This is made possible by its LRS (LC3 recognition sequence) located between the zinc finger and UBA domains of p62, where residues interact with the N-terminus and ubiquitin domain of LC3 [59,60]. Interestingly, knockout of p62 does not appear to markedly affect levels of ubiquitinated proteins in the cell, possibly due to compensatory action by Nbr1 (neighbor of BRCA1 gene 1), which interacts with LC3 in a similar manner [57,61]. Loss of function of Nbr1 leads to perturbation of p62 levels and hyperactivation of p38 MAPK that favors osteoblastogenesis [62]. Autophagy has been involved in hypoxia-induced osteoclast differentiation [63], and p62 may play a role in the starvation-induced autophagy in human osteoclasts [4].

#### p62 and molecular cross-talk between apoptosis and autophagy

The consequences of autophagy mainly favor cell survival, although a complex crosstalk exists between autophagy and apoptosis pathways [55]. While p62 has been involved in the selective autophagic degradation of many proteins, p62 is also involved in several apoptotic and survival pathways. RANKL stimulation contributes to osteoclast survival through p62-driven activation of NF-κB and MEK/ERK pathways [51]. Conversely, p62 also interacts with caspase-8, and is crucial for efficient caspase-8 activation by promoting aggregation of cullin-3 mediated-polyubiquitination [64]. On the other hand, p62 may be cleaved by caspase-6 and -8 in response to death receptor activation [65], and caspase-8, as well as p62, may be degraded by autophagy [66]. Thus, interrelationships exist between autophagy that affect the efficiency of apoptosis, and apoptosis that affects p62-dependent autophagy [55].

### Impact of PDB-associated p62 mutations on osteoclasts

Paget's disease of bone (PDB) is characterized by focal and disorganized increases in bone turnover, and primarily affects osteoclasts [67]. The role of p62 as an important modulator of bone turnover has first been highlighted by the discovery of mutations of the *SQSTM1 *gene in numerous pagetic patients, the p62^P392L ^substitution being the most frequent.

#### Impact of the p62^P392L ^mutation on osteoclast signaling

The significance of the p62^P392L ^mutation has been studied in a series of *in-vitro *experiments, using osteoclast precursors derived from the peripheral blood of PDB patients carrying the p62^P392L ^gene, and from bone marrow cells or cord blood monocytes (CBM) of normal subjects transfected with the wild-type p62 (p62^wt^) or the p62^P392L ^gene. These osteoclast precursors are hyper-responsive to osteoclastogenic factors, such as RANKL and TNFα, and the p62^P392L^-transfected cells have an enhanced ability to resorb bone [68]. Transgenic mice expressing the p62^P394L ^gene (p62^P392L ^equivalent in human) develop PDB-like bone lesions [69], as transgenic mice coexpressing the measles virus nucleocapsid (MVNP) under the TRAP promoter and knocked-in p62^P394L ^[70]. Previous findings have revealed that in human osteoclasts, even prior to RANKL stimulation, p62 associates with both activated PDK1 and PKCζ or activated PKCζ/λ in PDB osteoclasts, in osteoclasts from healthy donors harboring the p62^P392L ^gene, and in p62^P392L ^transfected osteoclasts [37]. Although both wild-type and mutated p62 over-expression favors the formation of activated PKCζ/λ-p62 complexes, only that of the mutated gene led to an increased basal level of NF-κB activation [37]. Similarly, overexpression of the p62^P392L ^mutant in HEK293 or Cos-1 cells increases basal and RANKL-induced NF-κB activation, more than overexpression of the wild-type p62 [71]. Finally, the presence of the PDB-associated p62^P392L ^mutation upregulates NFATc1 expression in pre-osteoclasts, favoring the increased osteoclastogenesis and osteoclast activity associated with metabolic bone disease [28,72]. These findings strongly suggest that p62^P392L ^contributes at least in part to the induction of an activated stage in osteoclasts by stimulating signaling pathways. More recent studies have further confirmed that CYLD knockdown significantly increased c-Fos expression in cells transduced to express both wild-type and mutant p62, without necessitating RANKL stimulation. Likewise, mutations to the UBA domain of p62 lead to a reduction in CYLD activity, and thus an increase in osteoclast development and resorption [72].

#### Impact of the p62^P392L ^mutation on protein turnover

All of the mutations identified to date in PDB patients are clustered either within or near the C-terminal region of the p62 protein that embodies the ubiquitin-associated (UBA) domain, and affect its interactions with multiubiquitin chains, suggesting that an alteration of ubiquitin-chain binding by p62 is important in the development of PDB [73,74]. The accumulation of p62-associated proteins, due to impaired degradation through proteosomal or autophagic pathways, could explain some aberrant cellular functions. As p62 is a key player in selective autophagy, and may have a role in the formation of inclusion bodies [53,57], and because the inclusion bodies found in Pagetic osteoclasts resemble those observed in diseases with defective autophagy, a dysregulation of the autophagy process may well be part of the pathogeny in PDB, although so far no direct evidence has been provided for its role in the phenotype of Pagetic osteoclast [75]. In addition, the p62^P392L ^mutation did not affect the p62-related aggregate formation in human osteoclasts [4].

#### Osteoclast apoptosis in PDB

In osteoclast cultures derived from PDB patients it has been observed that lower rates of apoptosis are induced by the deprivation of survival factors or by death inducers, such as TRAIL, Fas activating antibody or TGF-β [37]. In CBMs cultures, apoptosis rates are lower in osteoclasts overexpressing either p62^wt ^or p62^P392L ^than in controls. Furthermore, it has been shown that p62 is overexpressed in PDB osteoclasts, and this could contribute to their resistance to apoptosis, given that increased expression of p62 could in itself have a protective effect against cell death by preventing the build-up of potentially cell-damaging proteins [56,76]. In addition, the basal and RANKL-induced activation of NF-κB may influence survival in PDB osteoclasts. Finally, some genes involved in osteoclast apoptosis (encoding Caspase-3, and TRAIL-R1) are downregulated in Pagetic osteoclasts [77], and the expression of anti-apoptotic Bcl-2 gene is upregulated [78]. So, while it is clear that apoptosis is hindered in PDB osteoclasts, and that the presence of PDB mutations in the p62 gene is involved in this, the mechanisms by which these apoptotic pathways are altered are still unknown.

## Conclusions

Overproduction or changes in p62 activity has been shown to lead to impaired signaling and deficient autophagy in other cell and receptor models [57]. The probability remains that the same buildup of p62 and/or faulty UBA activity may be responsible for increased p62 substrate activity, and simultaneous reduced shuttling/ubiquitin-related activity, altering feedback and signaling loops. Since recent data also support the notion that p62 acts as a conformational adaptor, linking substrates from one domain to substrates bound to another, and not just the proteasome, p62 affects the cell in more ways than degradation and shuttling [5,79]. Given the number of mechanisms regulated either directly or indirectly by p62, its importance to osteoclast activation is unquestionable; playing an intimate role in protein signaling, polyubiquitination, and trafficking. The exact breadth of the influence of p62 in the osteoclast remains to be fully identified, and thus this RANKL regulator and its substrates provide targets of interest for exploration in order to better understand osteoclast activation signaling.

## Competing interests

The authors declare that they have no competing interests.

## Authors' contributions

SMcM and SR contributed equally in the writing of this manuscript. Each author read and approved the final manuscript.
